# Lifetime trajectories of male mating effort under reproductive conflict in a cooperatively breeding mammal

**DOI:** 10.1098/rspb.2024.1499

**Published:** 2024-09-18

**Authors:** Graham Birch, Hazel J. Nichols, Francis Mwanguhya, Faye J. Thompson, Michael A. Cant, Jonathan D. Blount

**Affiliations:** ^1^ Centre for Ecology & Conservation, Faculty of Environment, Science & Economy, University of Exeter, Penryn Campus, Cornwall TR10 9FE, UK; ^2^ Department of Biosciences, Swansea University, Singleton Campus, Swansea SA2 8PP, UK; ^3^ Banded Mongoose Research Project, Queen Elizabeth National Park, Kasese District Uganda

**Keywords:** life-history, reproductive conflict, cooperative breeder, restraint, reproductive costs, male

## Abstract

The costs of reproductive conflict can shape the evolution of life-histories in animal societies. These costs may change as individuals age and grow, and with within-group competition. Social costs of reproductive conflict have been invoked to explain why females might gain from delaying maturity or ceasing reproduction midway through life, but not in males. Here, we analyse more than 20 years of data to understand how individual male banded mongooses adjust their reproductive activity in response to the costs of reproductive conflict. In banded mongoose groups, multiple female breeders enter oestrus synchronously that are each guarded by a single male that aggressively wards-off rivals. The heaviest males in the group gained the greatest share of paternity. Those lighter males that are reproductively active paid disproportionate survival costs, and by engaging in reproductive activity early had lower lifetime reproductive success. Our results suggest that reproductive inactivity early in life is adaptive, as males recoup any lost fitness by first growing before engaging in less costly and more profitable reproductive activity later in life. These results suggest that resource holding potential of males and the intensity of reproductive conflict interact to shape lifetime schedules of reproductive behaviour.

## 1. Introduction

The assumption that reproduction involves costs to future fecundity and/or survival has formed the bedrock of life-history theory since its inception [1–8]. Owing to finite resources, individuals are expected to balance the costs and benefits of reproduction across their lifespan to maximize their lifetime reproductive success (LRS) [5–7]. These costs and benefits can vary according to an individual’s condition. Individuals in good condition might escape or reduce life-history trade-offs as they have more metabolic resources to put towards both reproductive effort and somatic maintenance [9,10]. The condition of individuals can change over time, for example as individuals grow they will have more resources to put towards reproductive effort, with individuals of high body mass typically the most fecund or successful competitors in a population [11,12].

In social groups, individuals can assume non-reproductive subordinate roles despite the cost of losing out on reproductive opportunities. Why these non-reproductive roles are assumed may reflect how the life-history trade-offs faced by these individuals change throughout their life. Younger individuals may not have acquired the resources either to successfully reproduce independently [13,14] or to reproduce without subjecting themselves to higher reproductive costs [15,16], and cooperative breeding groups can form when these individuals instead stay at home to help kin [14]. Non-breeding roles in social groups may allow these individuals to optimize their reproductive life-histories.

Rather than an ultimate assessment of the adaptive value of non-reproductive roles, previous hypotheses for why these roles exist typically focus on the proximate constraints on reproduction. For example, subordinates may face constraints in access to unrelated mating partners in their natal groups, and rather than inbreed, these individuals assume non-reproductive roles until unrelated mating partners become accessible (for example, as in female Damaraland molerats, *Cryptomys damarensis*) [17]. Where there are unrelated mating partners, subordinate reproduction may instead be directly suppressed by dominants through threats and violence [18–26], with the degree of suppression increasing with the level of reproductive skew. A single dominant pair may monopolize reproduction in despotic high skew groups compared with egalitarian lower skew groups where suppression is relatively weak and multiple same sex members reproduce [27,28]. Although we understand the proximate constraints on subordinate reproduction, there have been limited attempts to assess the measurable costs and benefits of adopting non-reproductive roles in these groups, without which we cannot fully understand the importance of adaptive life-history decisions in their emergence.

The life-histories of individuals in social groups may adaptively adjust to these changing reproductive constraints. Social constraints can include the condition of helpers and mating partners, with offspring having worse survival outcomes in groups with only genetically incompatible or low-quality mating partners, or in cooperative breeding groups with fewer helpers [22,29,30]. Here, we focus on the social constraint of reproductive conflict. Through suppression, higher relative resource holding potential (RHP) same-sex rivals increase the social costs, and reduce the likelihood of gaining fitness, for males that engage in reproductive conflict [27,31], defined in this study as pre-copulatory competition over mating partners. When suppression fails to prevent reproductive conflict, fights can be exceptionally costly in cooperative groups and can even escalate to the eviction of losers [32–35]. Such evictees lose the help and protection of the group for themselves and their potential offspring [36], and face an associated high failure rate for establishing new groups [29,37]. To avoid these social costs, young subordinates may withhold reproductive activity to wait in queues for future reproductive roles [32,37,38]. They may appease dominant individuals through submissive behaviours [39,40], by restricting growth to appear less of a threat [41,42], and by ‘paying to stay’ through cooperative behaviour such as offspring care [39]. As these individuals grow and gain in RHP relative to same-sex rivals the social costs of engaging in reproductive conflict should relax, and the reproductive roles adopted should adaptively adjust accordingly over the lifespan in such a way that may recoup any costs of delaying reproduction through more profitable and less costly reproduction later in life.

Examples demonstrating how life-histories adjust to the social costs of reproductive conflict have predominantly come from females [27,38,43,44]. For males in social groups, adjustments in reproductive life-histories have largely been described in response to ecological constraints. For example, African striped mice (*Rhabdomys pumilio*) delayed dispersal under high population densities [13], and in cooperative breeding birds [45,46] such as long-tailed tits (*Aegithalos caudatus*) males redirected helping effort to the clutches of kin when their own nests failed [47]. Much less is known about adjustments in male reproductive life-histories to the cost of reproductive conflict within social groups, despite the significant costs associated with reproductive competition among males [48–52]. In mammals, in particular, this knowledge gap may be explained by the less tractable nature of male reproductive life-histories in social groups. It is challenging to collect accurate long-term data when copulations are often difficult to observe compared with the relatively conspicuous changes in the morphology and behaviours of reproducing females, and it can be impossible to confirm paternity without the availability of genetic data. Additionally, many males in social groups are obligate dispersers, and large dispersal distances make it difficult to identify the past life-history of males that form or join non-natal groups. Where these problems can be overcome, how reproductive conflict alters the reproductive life-histories of males deserves assessment.

We use a comprehensive dataset spanning more than two decades, comprising behavioural, genetic and life-history data, to explore how reproductive activity in male banded mongooses adjusts to the costs and benefits of reproductive conflict. Banded mongooses live in large groups of up to 60 individuals, although groups of 10–30 are more typical (median = 18 adults, interquartile range = 9.25) [53,54]. The most common cause of adult mortality in our study population is predation, with disease or death attributed to senescence being less common [54]. Groups reproduce around four times per year [55]. Both sexes reach sexual maturity at around 1 year of age [56]. Same-sex banded mongoose group members are typically highly related to each other owing to budding dispersal—groups are formed from same-sex bands originating from the same group [54]. Banded mongooses are intermediate skew cooperative breeders with reproductive opportunities spread across a ‘core’ of breeding adults (1−5 females and 3−7 males) that reproduce 3−4 times per year [54], while other male group members remain reproductively inactive. All adult females in banded mongoose groups enter oestrus over a period of 7–10 days, preventing their monopolization by a single dominant male [53]. On average, older females enter oestrus first, followed by younger females a few days later [53]. The period from the first to last signs of oestrus within a group is labelled an ‘oestrus event’. Owing to the higher survival rate of males, and their tendency to stay in their natal group long-term compared with females, which are more regularly evicted [34,55], most groups have heavily male-biased sex ratios [53]. This skewed sex ratio sets the stage for reproductive conflict between males over a limited number of breeding females. During oestrus events, reproductive males guard individual females by following them closely and aggressively defending them from other males [53]. Guarding males remain active throughout the oestrus event, noticeably reducing foraging effort compared with inactive males. Older males tend to guard older, more fecund females, despite efforts by females to escape their guards [53].

Kin selection may play a role in adaptive adjustments to reproductive life-histories in social groups [27,57]. However, as males in banded mongoose groups are invariably closely related, this prevents comparisons between high- and low-relatedness social environments in our wild study system. Therefore, in this initial exploration into the reproductive life-histories of male banded mongooses, we focus on reproductive activity adjustments in accordance with direct reproductive costs and fitness benefits.

To test how male banded mongooses adjust their reproductive activity across their lifespan, we first employed state-transition models to ask whether there is evidence that males delay reproductive activity when they are at an RHP (weight and age) disadvantage compared with their competitors. Secondly, using the same models, we asked how males decline in reproductive activity as they senesce. We expected activity to decrease as RHP declines at the oldest age classes in line with previously identified weight declines in aged banded mongooses [58] and in other taxa [59]. We then assessed the degree to which paternity in each oestrus event is dominated by the highest RHP males in the group, and whether there is evidence that paternity declines rapidly in old age. We also asked whether males suffer energetic costs in the form of weight loss as a result of engaging in reproductive activity. Then, we asked whether the costs of reproductive activity contribute to mortality, and if low RHP males are at a higher risk of death. Finally, we asked how the timing with which a male first becomes reproductively active affects its LRS, expecting to find that males have higher LRS if they delay their reproductive activity until they increase in RHP compared with rivals. Simultaneous assessment of patterns of reproductive activity, mortality and reproductive pay-offs allowed us to judge whether the costs and benefits of reproductive activity adaptively shape the reproductive life-histories of male banded mongooses.

## 2. Methods

### (a) Study population

We collected data from a banded mongoose population living on the Mweya Peninsula, Queen Elizabeth National Park, Uganda (0°12′S, 29°54′E) between April 2003 and February 2021. For climate and habitat details, see Cant *et al*. [54]. Each individual was given a unique fur shave pattern on a small area of their back for identification. The history of each individual and group membership is known through life-history data collection ongoing since 1995 [54].

### (b) Long-term data collection

All long-term data collection has been previously described, which includes observations of the reproductive behaviour of males [11,53], life-history information [54], weights [58] and a genetic pedigree [60,61]. We briefly summarize these methods and data processing. For further details and clarifications on all long-term data collection methods and data processing, please see the electronic supplementary material accompanying this article.

#### (i) Reproductive behaviour

Since 2000, focals have been performed on females to record the reproductive behaviour they receive from males during each group oestrus event. Oestrus events are defined as the period spanning the start of oestrus until reproductive behaviour ceases. Male reproductive behaviour from all data collection days was summarized into one state for each oestrus event. Males that guarded on any day of the oestrus event were defined as guards, with the exception of cases where they were defined as sneaker males if they more often used opportunistic sneaky tactics. Males that had no observed reproductive activity on any day of an oestrus event were defined as inactive subordinates.

#### (ii) Life-history and weight data collection

All males in this analysis were followed from birth to death and are therefore of known age, and individual body weights have been collected from the population since 2000. To obtain a measure of relative RHP, males were ranked in age relative to other same-sex group members, and relative weight of each male compared with the average male member was calculated (group-centred weight: weight – mean group weight). Weight loss was calculated from pre- and post-oestrus weight.

#### (iii) Pedigree data

Genetic pedigrees have been collected since 2003. A banded mongoose’s gestation period is around nine weeks [53,62]. 105 group litters (402 offspring) could be connected to 180 sires in oestrus events approximately nine weeks prior to the litter’s birth date (ended 59 ± 15 days before).

### (c) Statistical models

All models were fitted using Bayesian inference (JAGS MCMC) in R [63]. To improve model convergence, numerical covariates with a range below 0 and above 1 were standardized. Posteriors were checked for multi-collinearity using the ggmcmc package [64]. Chain convergence was checked using rhat values from the JAGS model output, with all models showing convergence of chains for each fitted parameter (*R* < 1.1). Convergence was also checked using traceplots by eye (ggmcmc).

### (d) Mortality and state transition modelling

In this analysis, we aimed to focus on active mate guarding and inactive subordinate roles. Secondary reproductive males (sneakers) are present but were found to be rare for all values of age and weight (<0.10 transition probability). Modelled transitions to guarding or subordinate roles take account of the probability to transition to sneaking roles, but for simplicity, and because the costs and benefits of sneaking form the topic of a currently unpublished companion paper, model outputs for transitions to these sneaking roles have been omitted. Males must have been involved in at least two oestrus events for transition probabilities to be calculated, so males that only lived through a singular observed oestrus event are not included in these models.

The transition probability of males from one reproductive status to the next, and state-specific probability of dying before the next oestrus event, were modelled in relation to male age, age rank, and group-centred weight using JAGS MCMC [63,65]—the former testing for how reproductive activity is adjusted, and the latter to test for how costs of reproductive activity manifest throughout the lifespan of male banded mongooses. This model was based on 320 males undergoing 2999 transitions in total between four states (subordinate, sneaker, guard and dead). Using a state-transition matrix mortality (iterations = 50 000, thinning interval = 100, burn-in = 5000, chains = 3) and reproductive state transitions (iterations = 20 000, thinning interval = 100, burn-in = 2000, chains = 3) were modelled separately. See the electronic supplementary material for specification of the transition matrix.

To allow the assessment of a separate senescence effect while avoiding problems with multi-collinearity, state transitions and mortality models were run twice—first with age rank and second with quadratic age. Group-centred weight was included in each age model variation. Age rank and group-centred weight (*r* = 0.44), and age and group-centred weight (*r* = 0.37), were moderately correlated and did not produce problems with model convergence. Indeed, running separate models for moderately correlated variables may lead to bias exaggerating their significance [66]. Interactions between weight and age were, however, not considered since interactions between moderately correlated variables can produce significant multi-collinearity problems [66].

To control for common group membership during oestrus events and repeated sampling of the same males, random effects for oestrus event ID (*n* = 375), group ID (*n* = 20) and male ID (*n* = 320) were fitted for state transition models. To control for probability of death increasing with time, mortality transition models were also fitted with time to next oestrus event. To remove cases where excessive time had passed before death since the last oestrus event, data were truncated so that males that died more than a year from the last oestrus event were removed from analysis (random effects fitted: oestrus event ID (*n* = 371), historical group ID (*n* = 20) and male ID (*n* = 280)). Initially, mortality analysis included time interactions with all fixed effects, but these were later removed when none proved credible.

### (e) Oestrus event fitness models

To test for how each male’s share of a group’s paternity changes throughout its lifespan, the number of offspring sired by each male in a given oestrus event was regressed against group-centred weight with a binomial error structure (iterations = 20 000, thinning interval = 100, burn-in = 2000, chains = 3), with total offspring sired in an oestrus event used as the maximum number of successes. Mirroring the transition and mortality models, two model variations were run, one including age rank and the other quadratic age. To control for common group membership and repeated sampling on individuals, random effects for oestrus event ID (*n* = 105), historical group ID (*n* = 11) and male ID (*n* = 388) were fitted.

### (f) Weight loss models

554 weight changes from subordinates and 265 weight changes from guards were included. To control for common group membership during oestrus events and repeated sampling on the same males, random effects for oestrus event ID (*n* = 118), group ID (*n* = 20) and male ID (*n* = 259) were fitted. Percentage weight loss for each individual over an oestrus event was normally distributed, and as such was regressed with behavioural state (subordinate versus guard) using a Gaussian distribution using the JAGS MCMC engine (iterations = 20000, thinning interval = 100, burn-in = 2000, chains = 3). Initially, we wanted to assess whether age had an effect on weight loss for guards compared with subordinates. However, age, or age in interaction with state, was not fitted owing to issues with multi-collinearity with state.

### (g) First onset of reproductive activity and lifetime reproductive success models

229 males have recorded observations of reproductive activity. Lifetime number of offspring sired (LRS) by each of these males was zero-inflated (*n* = 95); therefore LRS was regressed using zero-inflated negative binomial models using the JAGS MCMC engine (iterations = 20 000, thinning interval = 100, burn-in = 2000, chains = 3). LRS was regressed against the age rank and group-centred weight of each male relative to other group members when they first engaged in reproductive activity (first observed as a guard). A second model regressed against absolute age and weight (not relative to other males’ age or weight in the same group) was also fitted. To control for common group membership, group ID was fitted as a random effect in both models (*n* = 15).

### (h) Model output processing

**Figure 1.**
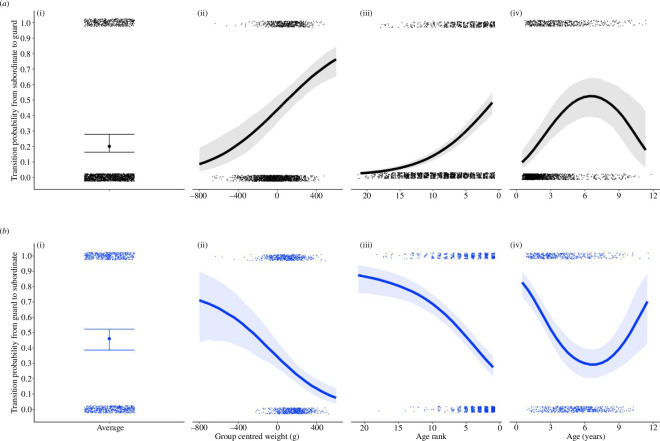
The effect of group-centred weight (ii), age rank (iii) and age (iv) on: (*a*) the probability of transitioning from a reproductively inactive subordinate state in one oestrus event to a reproductively active guard in the next event, and (*b*) the probability of transitioning from a reproductively active guard to an inactive subordinate. Transitions where males retain the same reproductive activity from one oestrus event into the next are approximately equal to 1 − the above transitions. Lines represent the mean posterior probability and ribbons are credible intervals. Black lines and grey ribbons correspond to transitions to an active guarding role, and blue lines and ribbons to transitions to an inactive subordinate role. Panel (i) shows mean (points) and credible intervals (standard error bars) for all four transitions for reference based on a null model with no covariates.

**Figure 2.**
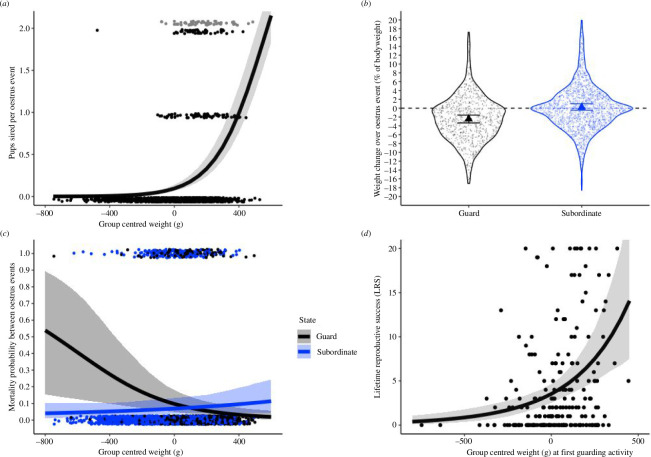
Predicted (*a*) mean number of pups sired per oestrus event given a male’s weight relative to other male group members; (*b*) weight change over an oestrus event given a male’s reproductive state; (*c*) mortality given a male’s reproductive state and weight relative to other group members; and (*d*) lifetime reproductive success given a male’s weight relative to other group members at first guarding activity. Black/grey represents (*b*) weight change and (*c*) mortality for guards, and blue represents weight change and mortality for subordinates. Single bold triangles represent the modelled mean weight change in (*b*), and error bar the upper and lower credible intervals. For all other plots, credible intervals are represented by ribbons (*a*,*c*,*d*). Points show raw data for all plots. For number of pups sired per oestrus event (*a*), grey dots represent cases where males sired more than two pups during the oestrus event. For weight change (*b*), the density distribution of raw weight change is represented by a violin plot and the dashed line represents neutral change (0).

For all models, significance table outputs were generated using the MCMCvis package [67], and model diagnostic plots using ggmcmc. Plotted probabilities were simulated from the posterior distribution extracted using the jagsUI package [68] (figures 1 and 2; electronic supplementary material, figure S2,S3,S4,S5).

## 3. Results

### (a) Patterns of reproductive activity throughout the lives of males in banded mongoose groups

On average, subordinates were more likely to stay as a subordinate in the next oestrus event (figure 1; mean = 0.72, high credible interval (hci) = 0.756, low credible interval (lci) = 0.644) than to transition into a guarding role (mean = 0.2, hci = 0.28, lci = 0.16), while the transition probabilities from a guarding role were not significantly different (figure 1; guard to subordinate—mean = 0.46, hci = 0.523, lci = 0.386; stay guard—mean = 0.41, hci = 0.5, lci = 0.34).

Overall, age rank and group-centred weight have similar independent effects on transition probabilities (figure 1(iii)). There was a significant linear effect, whereby as young mongooses moved up in weight and age rank in the group, they became more likely to gain a guarding position and less likely to stay in a subordinate position (electronic supplementary material, table S1, model 1a). Parity between the probabilities of staying as a subordinate compared with gaining a guarding role was reached at the oldest age ranks (1 and 2) and at around 50 g above the group average weight. At young age ranks and low weights, males rarely became a guard, for example subordinates at age rank 11, or 200 g below the mean weight of other males in the group, were predicted to gain a guarding role only *ca* 10% of the time (mean = 0.11, hci = 0.14, lci = 0.08 and mean = 0.09, hci = 0.13, lci = 0.07), tending towards 0 at younger age ranks and lighter weights. Predictions of age rank and weight diverge at cases of extreme weight, whereby age rank 1 subordinates were predicted to gain a guarding role around 50% of the time (mean = 0.48, hci = 0.55, lci = 0.41), while those of extreme weights (97.5% quantile + 320.4 g, max. = +494.48 g) had a higher predicted probability to become a guard, approaching two times out of three (weight = +450 g, mean = 0.67, hci = 0.77, lci = 0.58).

Similarly, there were independent significant linear effects of weight and age rank for keeping or losing a guarding role; heavier and older age rank males were more likely to keep a guarding position once obtained, and less likely to become an inactive subordinate (electronic supplementary material, table S1, model 1a). Parity for keeping as opposed to losing a guarding role was reached at age rank 4 and −150 g, younger and lighter than when parity was reached for staying compared with transitions out from a subordinate role (age rank 1 or 2 and +50 g). At the low end of probabilities, males had *ca* 10% probability of keeping their guarding position at age rank 12 and weight −250 g (mean = 0.1, hci = 0.16, lci = 0.05 and mean = 0.1, hci = 0.17, lci = 0.05). Guards of age rank older than 4 were more likely to hold than lose their guarding role with age rank 1 males, who as a subordinate would have had parity in their transition probabilities, having a 63% probability of keeping their guarding role (lci = 0.53, hci = 0.75). Guards of extreme weights (97.5% quantile = +382.59 g, max = +520.63 g) had a predicted probability of keeping a guarding role exceeding 80% (weight = +450, mean = 0.81, hci = 0.89, lci = 0.7), higher than predicted at the highest age ranks.

Age had a significant quadratic effect on all transition probabilities (electronic supplementary material, table S1, model 1b). The probability of gaining and keeping a guarding role mirrored the effect of age rank (figure 1*b*(iii)), with males tending to remain reproductively inactive well past sexual maturity (1 year) until reaching a peak of guarding at 6.5 years of age (subordinate to guard: mean = 0.57, hci = 0.67, lci = 0.48 and staying guard: mean = 0.65, hci = 0.75, lci = 0.56), after which probabilities of guarding decreased continuously as males aged. The switch point for the quadratic effect at 6.5 years was just below the mean age of age rank 1 males in oestrus events (mean = 7.25, interquartile range (IQR) = 3.25), suggesting an ageing effect was masked in age rank 1 males by groups that lack older males. As the probability of gaining or holding a guarding position trended down with age, parity between transitions to a subordinate and guarding role was reached at around 8.5 years (from subordinate) and 9.5 years (from guarding) (figure 1). At extreme ages (subordinate: 97.5% quantile = 10.24, maximum = 11.8; guard: 97.5% quantile = 10.13 years, maximum = 11.36 years), transition probabilities to gain or keep a guarding role were as low as 20 and 30%, respectively (subordinate to guard: age = 10.5 years, mean = 0.19, hci = 0.37, lci = 0.09; stay guard: age = 10.5 years, mean = 0.3, hci = 0.53, lci = 0.13). Beyond 8 years of age, increasing age corresponded to a decrease in weight of males (electronic supplementary material, figure S1), which may correspond with the decreased probability to gain and keep a guarding role at extreme ages and low group-centred weights. A linear decline past the quadratic switch points was further verified by running separate models on a truncated dataset (see electronic supplementary material).

### (b) Distribution of siring success in banded mongoose groups

The probability to maintain or transition into guarding roles reflected the probability of siring offspring. Older age rank and heavier males were significantly more likely to sire a given pup (electronic supplementary material, table S1, model 2a, figure S2b,c), while age had a significant negative quadratic effect (electronic supplementary material, tables S1 and S2b) with a decline in the probability of siring at old ages, past 7 years old (for more detail, see electronic supplementary material, figure S2d). Extremely heavy males were predicted to sire more pups beyond that predicted by the oldest age ranks, with mongooses weighing at least 400 g more than the average male in the group siring at least 1 pup per oestrus event (400 g: mean = 1.00, hci = 1.35, lci = 0.76; 450 g: mean = 1.25, hci = 1.64, lci = 0.98).

### (c) Weight loss costs of reproductive activity

The mean observed weight for a male mongoose was 1455.99 ± 4.64 g s.e.m. Guards lost on average 2.36% (figure 2; hci = −1.46%, lci = −3.33%) of their body weight, significantly more, 2.63% (*ca* 40 g), than subordinates (electronic supplementary material, table S1, model 3), for which net change was not significantly different from 0 (figure 2; mean = +0.21% , hci = +0.92%, lci = −0.53% — posterior effect overlaps 0).

### (d) Mortality costs of reproductive activity

Mean survival between oestrus events was not on average significantly different between guards (mean = 0.075, hci = 0.14, lci = 0.40) and subordinates (mean = 0.71, hci = 0.131, lci = 0.039) (electronic supplementary material, figure S3a). There was a significant negative effect of group-centred weight on mortality probabilities for guards but not subordinates (electronic supplementary material, table S1, models 4a, 4b). Below the average weight of males, guards started to have a higher mortality probability than subordinates (figure 2), although only at particularly low weights was this difference statistically significant (−450 g guard: mean = 0.3, hci = 0.60, lci = 0.10; −450 g subordinate: mean = 0.05, hci = 0.10, lci = 0.02). The mortality cost in low-weight guards was reflected in the raw data; relative to the weight of other males in the group, 29/200 (14.5%) guards of below average weight, and 2/908 (8%) of average and above weight, died before the next oestrus event. In contrast, 80/1190 (6.7%) subordinates of below average weight, similar to 80/976 (8.2%) of average and above weight, died before the next oestrus event. There was a similar negative effect of absolute weight on the mortality probability of guards and not subordinates (electronic supplementary material, table S2, model 4c).

Mortality increased as mongooses moved into older age ranks (electronic supplementary material, table S1). When simulating mortality probabilities, the effects of each state overlapped at all age rank values (electronic supplementary material, figure S3c). When simulated overall, increases in mortality with age rank were small, with a <5% increase in mortality from the youngest to the oldest age ranks for subordinates and negligible increase for guards (electronic supplementary material, figure S3c). There was no significant quadratic effect of age on mortality, and the linear effect of age mirrored age rank (electronic supplementary material, table S1 and figure S3d).

### (e) Effect of delaying reproductive activity on lifetime reproductive success

Absolute age and weight at first reproductive activity had a significant effect on LRS (electronic supplementary material, table S1, model 5b). LRS increased as males delayed their first guarding activity to older ages or higher weights (electronic supplementary material, figure S4*b*,*d*). However, when the competitive environment in groups is taken into account by fitting age rank and group-centred weight, only group-centred weight at first reproductive activity significantly affected LRS (electronic supplementary material, table S1, model 5a, figure S4a,c), with LRS increasing as males delayed reproductive activity until they reached higher weights relative to same-sex rivals (figure 2). For example, males that delayed reproductive activity until they were 250 g heavier than the average group member had a predicted LRS of 7.52 (hci = 10.93, lci = 4.98). This was higher than for males that delayed activity until they reached the average male group weight (mean = 3.53, hci = 4.65, lci−2.51), and was higher again than for males that started guarding at 350 g below the average male group weight (mean = 1.29, hci = 2.33, lci = 0.65).

## 4. Discussion

We found that males largely delayed reproductive activity until they became older and heavier relative to rival groups members. The oldest and the heaviest male intragroup competitors have the highest probability to guard. Males that reached old ages (associated with weight decline) became less reproductively active, suggesting a senescent effect. Paternity share mirrored reproductive activity in the group, with the heaviest and oldest males in groups dominating paternity share of group litters. Reproductive activity did impose significant costs on male banded mongooses. Reproductively active guards lost weight compared with inactive subordinates, which on average had no change in weight over the oestrus period. Mortality costs of reproductive activity were the greatest for lower-weight males in banded mongoose groups, those at a RHP disadvantage to rivals. Finally, males that delayed reproductive activity until they reached heavier weights compared with same-sex competitors increased their LRS. This points to the likelihood that the reproductive inactivity we have identified in low RHP males may be adaptive, as the costs of reproductive conflict with superior rivals are prohibitive and any lost reproductive fitness is recouped later in life as males grow.

Our investigation revealed adjustments in the reproductive activity of male banded mongooses consistent with reproductive queues. Males that were lighter relative to other males in the group delayed reproductive activity despite being sexually mature, staying in inactive subordinate roles. Social costs of competing with heavy males may explain why lighter males avoid reproductive activity. The high RHP individuals that dominate reproductive roles in social groups often suppress subordinate reproduction through regular threat displays or violence [25,53,69,70]. Yet, in male banded mongooses, outside of guarding females during short oestrus events, males are rarely observed threatening or attacking other male group members, suggesting a permanent hierarchy does not need to be enforced by dominant males. When low RHP males do engage in reproductive activity, they may suffer larger social costs owing to a competitive disadvantage in fights, which may have contributed to the higher mortality costs of reproductive activity at low weight relative to other group members. Restraint when at a competitive disadvantage likely serves to avoid social costs of reproductive conflict. These males may be passively coerced into reproductive restraint consistent with previously identified ‘hidden threats’, where escalation to direct suppression or violent punishment by dominants is rarely seen because subordinate transgression is rare [71]. For example, experimental manipulation revealed subordinate female banded mongooses that breed asynchronously with dominants are punished by infanticide—rarely seen under natural circumstances because females almost always breed synchronously [71]. We suggest low RHP males may similarly abstain from reproductive activity to avoid aggression by dominants. Dominant males may gain from ‘hidden threats’ as these reduce the need to actively suppress subordinate reproduction, which can be costly for dominants, for example females that carried out evictions in banded mongoose groups reduced their future fecundity [72]. If these ‘hidden threats’ are not respected by low RHP males, they may suffer high social costs as a result of reproductive conflict, as suggested by our mortality results. Instead of incurring social costs, low RHP males may typically choose to stay reproductively inactive while continuing to gain inclusive fitness by helping relatives [73,74].

Our finding of weight loss costs aligns with those previously reported [49], such as in reindeer (*Rangier tarandus*) during rutts [75], and other cases of mate-guarding such as in *Sceloporus virgatus* lizards [76]. The weight loss observed in our study is likely a result of the energetic demands of actively guarding females and the time spent away from foraging activities, as suggested by other studies [49,77]. Mortality costs of reproductive activity also increased as the relative weight compared with other males in the group decreased, yet only at below average weights were guards predicted to die between oestrus events more often than subordinates. These low RHP mortality costs could be explained by mismatched fights with superior rivals, but they could also indicate condition-dependent costs of reproductive activity [9,10]. Condition-dependent costs align with past evidence in other systems of higher mortality costs [6,16,78] or long-term fecundity costs [13,52] of reproductive activity in lower condition breeders. The low-weight mortality cost of reproductive activity in male banded mongooses mirrors the rarity with which males gain or maintain active guarding roles at low weight in our state transitional analysis. It also aligns with reduced LRS for males that begin reproductive activity at low weight. These results together suggest avoiding the costs of reproductive conflict adaptively shapes the reproductive life-histories of males in banded mongoose groups.

Condition-dependent costs would suggest that individuals do not face the same constraints for life-history trade-offs when they are in sufficiently good condition [7,79]. Instead of trade-offs, positive relationships between reproductive demands and survival can be observed as individuals that typically invest more into reproduction are in higher condition [80]. For example, survival in *Iberolacerta cyreni* lizards had a positive rather than a negative association with high reproductive activity [81]. Similarly, longevity was associated with ovarian function in *Bombus terretris* honeybees [82]. Without experimental manipulation or control of condition, variation in individual condition can often mask the costs of reproduction [83]. Survival costs may have only been detectable in our system owing to the longevity of our study, allowing relatively rare cases of constrained low-weight males engaging in reproductive activity to occur. Overall, reproductive costs in banded mongooses may follow the ‘big house, big car’ analogy [9,10,80], where lighter males cannot afford the costs of reproductive activity compared with heavier malesthat have accumulated enough wealth in terms of resources to afford the investment.

Reproductive inactivity may avoid delaying the growth of males in order to effectively fight in the future and ultimately secure reproductive success. For example, the energetic demands of early roaming in male African striped mice (*R. pumilio*) reduced LRS by delaying the age at which males acquired their own harems [51,52]. Similarly, the weight lost by guarding early in banded mongoose groups may delay the weight gain necessary to more successfully compete for guarding roles in future oestrus events, suggesting early reproductive activity could have latent fecundity costs together with the short-term survival costs we have found. Therefore, as well as avoiding social costs of reproductive conflict in the short term, the decision of young growing males to stay inactive in queues may be reinforced by the long-term need to gain weight to successfully challenge in future oestrus events. Ultimately, our LRS results suggest fitness lost owing to reproductive inactivity early on is recouped when males become reproductively active once obtaining these heavier weights compared with rivals.

Female choice may have played a role in the reproductive inactivity of males. Females can increase their reproductive fitness through choice by biasing fertilization to higher-quality fathers [84,85]. RHP is a common characteristic females use in mate choice as their sons stand to inherit their father’s competitiveness to continue to pass on their mother’s genes [86,87]. By guarding, males may reduce the ability of females to make their own mate choices. Yet, there is evidence that female banded mongooses retain some autonomy over mate choice in inbreeding avoidance [61], which may carry over to RHP-based mate choices. Avoidance by females could add an additional barrier to the success of low RHP males on top of reproductive conflict with superior rivals, which may further favour reproductive inactivity to avoid unnecessary reproductive costs for a small chance of success.

Exceptionally heavy males were the most reproductively active, above activity explained by age rank. The large condition advantage should be beneficial when considering the long-term endurance of consecutive bouts of reproductive activity. Banded mongooses reproduce throughout the year, with a new oestrus event often occurring two months after the last, soon after pups have emerged from the den [55], or more frequently where litters are unsuccessful. Frequent reproductive bouts mean that males must strive to recover rapidly from the costs of previous reproductive activity, which may lead to exhaustion going into the next reproductive bout if they have not replenished their condition in the interim period. Lower condition males should suffer more from reproductive exhaustion owing to having fewer resources to recover from previous reproductive activity. For example, experimental manipulations of the mating history of mosquito-fish (*Gambusia affinis*) revealed ageing males are unable to maintain sperm count and velocity compared with younger males during repeated bouts of reproduction [88]. Additionally, as the weight advantage a male has over rivals increases, it may better maintain an RHP advantage into future oestrus events to more easily secure guarding positions in the long term despite repeated episodes of weight loss.

As condition declined with age in senescing males, the probability they maintained reproductive activity, or sired offspring, similarly declined. As their RHP declines, these males may become vulnerable to displacement by rivals, aligning with senescence-associated exiting of reproductive roles in other animal societies. For example, Seba’s short-tailed bats (*Carollia perspicillata*) show a decline in the ability to maintain harems as males pass 5 years of age [65]. Also, senescing males’ reluctance to engage in reproductive activity may persist owing to the threat of social conflict with higher RHP rivals. However, when so close to the end of life, abstaining from costly reproductive activity may not contribute significantly to their LRS in the future. In fact, according to terminal investment theory, senescing individuals should invest maximally in reproduction when it becomes clear that there are few remaining reproductive opportunities [89]. Additionally, many intermediate-weight males relative to rivals remain inactive despite no significant difference in mortality costs between activity and inactivity found, which may appear suboptimal for their lifetime reproductive fitness. These seemingly cautious approaches to reproductive activity may only make sense when considering kin selection. Rivals are fellow productive group members and often close kin [90], so costs imposed on them due to reproductive conflict may reduce inclusive fitness.

The costs incurred through internal conflict may weaken the group as a whole. Weight loss and injuries due to conflict may mean members are not able to effectively contribute to cooperative behaviours such as offspring care, protection against predators, or fighting during intergroup wars [36]. Reduced contributions of individuals may reduce the survival and reproductive success of all group members. As such, models of the evolution of cooperative breeders centre around how conflict has been minimized [27]. Two alternative evolutionary pathways may minimize conflict and are characterized by mechanisms that are difficult to disentangle: suppression and voluntary restraint.

One pathway we have discussed is the suppression of conflict by dominants through imposing social costs on subordinates. For example in social insects, queens police reproduction in workers by promoting aggression and the consumption of the eggs of any females that attempt to reproduce [91,92]. The second pathway is kin selection, where individuals reduce reproductive conflict in order to help relatives for inclusive fitness benefits [27]. If kin-selected benefits are high, voluntary restraint may be selected without the need for suppression by dominants. For example, one experimental study found policing in carpenter ants (*Camponotus floridanus*) was not present in incipient colonies [93]. Worker reproduction is highly damaging to incipient colonies owing to a lack of caring capacity, and policing was suggested not to be necessary in these cases, as workers are under high kin selection to express voluntary reproductive restraint and help care instead of reproduce themselves [93]. Without such carefully designed experiments, inactivity to avoid the social costs of conflict and voluntary inactivity for kin-selected benefits are difficult to tease apart. Suppression may mean conflict is present but not observed unless subordinates transgress, which may only rarely be observable in natural systems. This gives the appearance of voluntary reproductive inactivity, such as if subordinates use cues to avoid conflict, for example pheromones or a queen’s cuticular hydrocarbon (CHC) profile in social insects [91,94]. We cannot tease apart suppression versus voluntary inactivity in our wild study system, but voluntary restraint could play a role in banded mongoose groups, which often have overlapping generations of related males [55]. Reducing kin-selected costs of internal conflict may serve as a significant selection pressure reinforcing the prevalent reproductive inactivity observed among mature males in this study.

The reduction in internal conflict among related males in banded mongoose groups is consistent with recent ideas suggesting a key step in evolutionary transitions to higher levels of social organization is the breaking of life-history trade-offs (i.e. major evolutionary transitions) [95]. Kin selection may favour low condition males to remain inactive in order to allow higher condition males to reproduce, which face a weakened trade-off between reproduction and survival. Kin selection could also favour reduction in the social costs of internal conflict to improve the fighting ability of the group in intergroup wars, as males specifically are important in determining the outcome of conflict [58]. Of course as discussed, the reproductive inactivity of lower condition mature males we have found may simply be a result of competitive displacement from reproductive positions, or ultimately self-interested attempts by subordinates to avoid the social costs of conflict in the group, which does not require kin selection to play a role. In our wild population, males are typically closely related to rival same-sex group members [55]. Without many social groups with non-related rivals as a comparison with related rivals, it will be difficult to disentangle how self-interested avoidance of social costs and kin selection mould the reproductive life-histories of male banded mongooses. Experimentally manipulated group demographics are not always possible when monitoring long-term projects, as in our case, but where they are possible comparisons of reproductive activity trajectories could allow us to understand the role kin selection plays moulding the life-histories of males in social groups.

## 5. Conclusion

The life-histories of male banded mongooses are shaped by the benefits of reducing costly reproductive conflict. Lower condition males remain inactive, which likely serves to avoid suffering higher social costs of reproductive activity, supported by higher mortality costs found for lower-weight males. Early reproductive inactivity may allow males to recoup lost fitness through more successful attempts to compete with rivals in the future, as suggested by their LRS. These costs and benefits fit well with the queueing dynamics we have found, suggesting young, lighter males adaptively delay reproductive activity. As males grow, the social costs of reproductive conflict decline, and increased condition may make the energetic demands of guarding easier to bear. Reduction in the costs of conflict is reflected in more consistent engagement in reproductive activity at older, but not senescent, age classes. Kin-selected benefits may play a role reducing reproductive conflict within banded mongoose groups. Disentangling how self-interested avoidance of social costs and kin selection mould the reproductive life-histories of males in social groups requires future research.

## Data Availability

All code and derived data for analyses is deposited on Dryad [96]. Supplementary material is available online [97].

## References

[1] Monaghan P , Metcalfe NB , Torres R . 2009 Oxidative stress as a mediator of life history trade-offs: mechanisms, measurements and interpretation. Ecol. Lett. **12** , 75–92. (10.1111/j.1461-0248.2008.01258.x) 19016828

[2] Speakman JR , Garratt M . 2014 Oxidative stress as a cost of reproduction: beyond the simplistic trade‐off model. Bioessays **36** , 93–106. (10.1002/bies.201300108) 24285005

[3] Zhang Y , Hood WR . 2016 Current versus future reproduction and longevity: a re-evaluation of predictions and mechanisms. J. Exp. Biol. **219** , 3177–3189. (10.1242/jeb.132183) 27802148 PMC5091378

[4] Harshman LG , Zera AJ . 2007 The cost of reproduction: the devil in the details. Trends Ecol. Evol. **22** , 80–86. (10.1016/j.tree.2006.10.008) 17056152

[5] Cody ML . 1966 A general theory of clutch size. Evolution **20** , 174. (10.2307/2406571) 28563630

[6] Kirkwood TBL , Rose MR . 1991 Evolution of senescence: late survival sacrificed for reproduction. Phil. Trans. R. Soc. Lond. B **332** , 15–24. (10.1098/rstb.1991.0028) 1677205

[7] Stearns SC . 1992 The evolution of life-histories. Oxford, UK: Oxford University Press. (10.2307/5403)

[8] Pianka ER , Parker WS . 1975 Age-specific reproductive tactics. Am. Nat. **109** , 453–464. (10.1086/283013)

[9] Reznick D , Nunney L , Tessier A . 2000 Big houses, big cars, superfleas and the costs of reproduction. Trends Ecol. Evol. **15** , 421–425. (10.1016/S0169-5347(00)01941-8) 10998520

[10] Metcalfe NB , Monaghan P . 2013 Does reproduction cause oxidative stress? An open question. Trends Ecol. Evol. **28** , 347–350. (10.1016/j.tree.2013.01.015) 23485157

[11] Nichols HJ , Amos W , Cant MA , Bell MBV , Hodge SJ . 2010 Top males gain high reproductive success by guarding more successful females in a cooperatively breeding mongoose. Anim. Behav. **80** , 649–657. (10.1016/j.anbehav.2010.06.025)

[12] Groenewoud F , Clutton-Brock T . 2021 Meerkat helpers buffer the detrimental effects of adverse environmental conditions on fecundity, growth and survival. J. Anim. Ecol. **90** , 641–652. (10.1111/1365-2656.13396) 33241582

[13] Schradin C , Lindholm AK , Johannesen J , Schoepf I , Yuen CH , König B , Pillay N . 2012 Social flexibility and social evolution in mammals: a case study of the African striped mouse (Rhabdomys pumilio). Mol. Ecol. **21** , 541–553. (10.1111/j.1365-294X.2011.05256.x) 21883591

[14] Cant MA . 2012 Cooperative breeding systems. In The evolution of parental care (eds NJ Royle , PT Smiseth ), pp. 206–220. Oxford, UK: Oxford Univesity Press. (10.1093/acprof:oso/9780199692576.003.0012)

[15] Lemaître JF , Gaillard JM , Pemberton JM , Clutton-Brock TH , Nussey DH . 2014 Early life expenditure in sexual competition is associated with increased reproductive senescence in male red deer. Proc. R. Soc. B **281** , 20140792. (10.1098/rspb.2014.0792) PMC4150313 25122226

[16] Lemaître JF , Berger V , Bonenfant C , Douhard M , Gamelon M , Plard F , Gaillard JM . 2015 Early-late life trade-offs and the evolution of ageing in the wild. Proc. R. Soc. B **282** , 20150209. (10.1098/rspb.2015.0209) PMC4426628 25833848

[17] Cooney R , Bennett NC . 2000 Inbreeding avoidance and reproductive skew in a cooperative mammal. Proc. R. Soc. Lond. B **267** , 801–806. (10.1098/rspb.2000.1074) PMC1690596 10819150

[18] Holmes MM , Goldman BD , Goldman SL , Seney ML , Forger NG . 2009 Neuroendocrinology and sexual differentiation in eusocial mammals. Front. Neuroendocrinol. **30** , 519–533. (10.1016/j.yfrne.2009.04.010) 19416733 PMC2748139

[19] Faulkes CG , Abbott DH , Jarvis JUM . 1991 Social suppression of reproduction in male naked mole-rats, Heterocephalus glaber. J. Reprod. Fertil. **91** , 593–604. (10.1530/jrf.0.0910593) 2013881

[20] Zhou S , Holmes MM , Forger NG , Goldman BD , Lovern MB , Caraty A , Kalló I , Faulkes CG , Coen CW . 2013 Socially regulated reproductive development: analysis of GnRh-1 and kisspeptin neuronal systems in cooperatively breeding naked mole-rats (Heterocephalus glaber). J. Comp. Neurol. **521** , 3003–3029. (10.1002/cne.23327) 23504961

[21] Swift-Gallant A , Mo K , Peragine DE , Monks DA , Holmes MM . 2015 Removal of reproductive suppression reveals latent sex differences in brain steroid hormone receptors in naked mole-rats, Heterocephalus glaber. Biol. Sex Differ. **6** , 31. (10.1186/s13293-015-0050-x) 26693002 PMC4676092

[22] O’Riain MJ , Bennett NC , Brotherton PNM , McIlrath G , Clutton-Brock TH . 2000 Reproductive suppression and inbreeding avoidance in wild populations of co-operatively breeding meerkats (Suricata suricatta). Behav. Ecol. Sociobiol. **48** , 471–477. (10.1007/s002650000249)

[23] Carlson AA , Young AJ , Russell AF , Bennett NC , McNeilly AS , Clutton-Brock T . 2004 Hormonal correlates of dominance in meerkats (Suricata suricatta). Horm. Behav. **46** , 141–150. (10.1016/j.yhbeh.2004.01.009) 15256303

[24] Montgomery TM , Pendleton EL , Smith JE . 2018 Physiological mechanisms mediating patterns of reproductive suppression and alloparental care in cooperatively breeding carnivores. Physiol. Behav. **193** , 167–178. (10.1016/j.physbeh.2017.11.006) 29730040

[25] Thompson FJ , Donaldson L , Johnstone RA , Field J , Cant MA . 2014 Dominant aggression as a deterrent signal in paper wasps. Behav. Ecol. **25** , 706–715. (10.1093/beheco/aru063)

[26] Smith JM , Harper D . 2003 Animal signals. Oxford, UK: Oxford University Press.

[27] Taborsky M , Cant MA , Komdeur J . 2021 Conflict. In The evolution of social behaviour, pp. 67–135. Cambridge, UK: Cambridge University Press. (10.1017/9780511894794.005)

[28] Ross CT , Hooper PL , Smith JE , Jaeggi AV , Alden E , Gavrilets S . 2023 Reproductive inequality in humans and other mammals. Anthropology **120** , 1–12. (10.1073/pnas.222012412) PMC10235947 37216525

[29] Doerr ED , Doerr VAJ . 2007 Positive effects of helpers on reproductive success in the brown treecreeper and the general importance of future benefits. J. Anim. Ecol. **76** , 966–976. (10.1111/j.1365-2656.2007.01280.x) 17714275

[30] Gilchrist JS . 2006 Reproductive success in a low skew, communal breeding mammal: the banded mongoose, Mungos mungo. Behav. Ecol. Sociobiol. **60** , 854–863. (10.1007/s00265-006-0229-6)

[31] Sato Y , Rühr PT , Schmitz H , Egas M , Blanke A . 2016 Age-dependent male mating tactics in a spider mite—a life-history perspective. Ecol. Evol. **6** , 7367–7374. (10.1002/ece3.2489) 28725404 PMC5513254

[32] Fitzgerald LM *et al* . 2022 Rank change and growth within social hierarchies of the orange clownfish, Amphiprion percula. Mar. Biol. **169** , 128. (10.1007/s00227-022-04117-9)

[33] Stephens PA , Russell AF , Young AJ , Sutherland WJ , Clutton-Brock TH . 2005 Dispersal, eviction, and conflict in meerkats (Suricata suricatta): an evolutionarily stable strategy model. Am. Nat. **165** , 120–135. (10.1086/426597) 15729644

[34] Cant MA , Hodge SJ , Bell MBV , Gilchrist JS , Nichols HJ . 2010 Reproductive control via eviction (but not the threat of eviction) in banded mongooses. Proc. R. Soc. B **277** , 2219–2226. (10.1098/rspb.2009.2097) PMC2880142 20236979

[35] Iwasa Y , Yamaguchi S . 2022 On the role of eviction in group living sex changers. Behav. Ecol. Sociobiol. **76** , 49. (10.1007/s00265-022-03159-9)

[36] Reeve HK , Hölldobler B . 2007 The emergence of a superorganism through intergroup competition. Proc. Natl Acad. Sci. USA **104** , 9736–9740. (10.1073/pnas.0703466104) 17517608 PMC1887545

[37] Cant MA , English S . 2006 Stable group size in cooperative breeders: the role of inheritance and reproductive skew. Behav. Ecol. **17** , 560–568. (10.1093/beheco/arj065)

[38] Bridge C , Field J . 2007 Queuing for dominance: gerontocracy and queue-jumping in the hover wasp Liostenogaster flavolineata. Behav. Ecol. Sociobiol. **61** , 1253–1259. (10.1007/s00265-007-0355-9)

[39] Bergmüller R , Taborsky M . 2005 Experimental manipulation of helping in a cooperative breeder: helpers ‘pay to stay’ by pre-emptive appeasement. Anim. Behav. **69** , 19–28. (10.1016/j.anbehav.2004.05.009)

[40] Reddon AR , Ruberto T , Reader SM . 2021 Submission signals in animal groups. Behaviour **159** , 1–20. (10.1163/1568539X-bja10125)

[41] Wong MYL , Buston PM , Munday PL , Jones GP . 2007 The threat of punishment enforces peaceful cooperation and stabilizes queues in a coral-reef fish. Proc. R. Soc. B **274** , 1093–1099. (10.1098/rspb.2006.0284) PMC2124475 17301018

[42] Hamilton IM , Heg D . 2008 Sex differences in the effect of social status on the growth of subordinates in a co‐operatively breeding cichlid. J. Fish Biol. **72** , 1079–1088. (10.1111/j.1095-8649.2007.01787.x)

[43] Croft DP *et al* . 2017 Reproductive conflict and the evolution of menopause in killer whales. Curr. Biol. **27** , 298–304. (10.1016/j.cub.2016.12.015) 28089514

[44] Lahdenperä M , Gillespie DOS , Lummaa V , Russell AF . 2012 Severe intergenerational reproductive conflict and the evolution of menopause. Ecol. Lett. **15** , 1283–1290. (10.1111/j.1461-0248.2012.01851.x) 22913671

[45] Hatchwell BJ . 2009 The evolution of cooperative breeding in birds: kinship, dispersal and life history. Phil. Trans. R. Soc. B **364** , 3217–3227. (10.1098/rstb.2009.0109) 19805429 PMC2781872

[46] Kingma SA , Bebbington K , Teunissen N , Peters A , Komdeur J . 2021 The evolution of delayed dispersal and different routes to breeding in social birds. Adv. Study Behav. **53** , 163–224. (10.1016/bs.asb.2021.03.003)

[47] Leedale AE , Lachlan RF , Robinson EJH , Hatchwell BJ . 2020 Helping decisions and kin recognition in long-tailed tits: is call similarity used to direct help towards kin? Phil. Trans. R. Soc. B **375** , 20190565. (10.1098/rstb.2019.0565) 32420850 PMC7331009

[48] Friesen CR , de Graaf SP , Olsson M . 2019 The relationship of body condition, superoxide dismutase, and superoxide with sperm performance. Behav. Ecol. **30** , 1351–1363. (10.1093/beheco/arz086)

[49] Ord TJ . 2021 Costs of territoriality: a review of hypotheses, meta-analysis, and field study. Oecologia **197** , 615–631. (10.1007/s00442-021-05068-6) 34716493

[50] Sharick JT , Vazquez‐Medina JP , Ortiz RM , Crocker DE . 2015 Oxidative stress is a potential cost of breeding in male and female northern elephant seals. Funct. Ecol. **29** , 367–376. (10.1111/1365-2435.12330) 25983364 PMC4429057

[51] Rimbach R , Blanc S , Zahariev A , Pillay N , Schradin C . 2019 Daily energy expenditure of males following alternative reproductive tactics: solitary roamers spend more energy than group-living males. Physiol. Behav. **199** , 359–365. (10.1016/j.physbeh.2018.12.003) 30521878

[52] Kanyile SN , Pillay N , Schradin C . 2021 Bachelor groups form due to individual choices or environmental disrupters in African striped mice. Anim. Behav. **182** , 135–143. (10.1016/j.anbehav.2021.10.005)

[53] Cant MA . 2000 Social control of reproduction in banded mongooses. Anim. Behav. **59** , 147–158. (10.1006/anbe.1999.1279) 10640376

[54] Cant MA , Vitikainen E , Nichols HJ . 2013 Demography and social evolution of banded mongooses. Adv. Study Behav. **45** , 407–445. (10.1016/B978-0-12-407186-5.00006-9)

[55] Cant MA , Nichols HJ , Thompson FJ , Vitikainen E . 2016 Banded mongooses: demography, life history, and social behavior. In Cooperative breeding in vertebrates: studies of ecology, evolution, and behavior (eds WG Koenig , JL Dickinson ), pp. 318–337. Cambridge, UK: Cambridge University Press. (10.1017/CBO9781107338357.019)

[56] Vitikainen EIK , Thompson FJ , Marshall HH , Cant MA , Cant MA . 2019 Live long and prosper: durable benefits of early-life care in banded mongooses. Phil. Trans. R. Soc. B **374** , 20180114. (10.1098/rstb.2018.0114) 30966878 PMC6460079

[57] Johnstone RA , Cant MA . 2010 The evolution of menopause in cetaceans and humans: the role of demography. Proc. R. Soc. B **277** , 3765–3771. (10.1098/rspb.2010.0988) PMC2992708 20591868

[58] Green PA , Thompson FJ , Cant MA . 2022 Fighting force and experience combine to determine contest success in a warlike mammal. Proc. Natl Acad. Sci. USA **119** , e2119176119. (10.1073/pnas.2119176119) 35700363 PMC9231503

[59] Fasel NJ , Wesseling C , Fernandez AA , Vallat A , Glauser G , Helfenstein F , Richner H . 2017 Alternative reproductive tactics, sperm mobility and oxidative stress in Carollia perspicillata (Seba’s short-tailed bat). Behav. Ecol. Sociobiol. **71** , 11. (10.1007/s00265-016-2251-7)

[60] Nichols HJ , Cant MA , Hoffman JI , Sanderson JL . 2014 Evidence for frequent incest in a cooperatively breeding mammal. Biol. Lett. **10** , 20140898. (10.1098/rsbl.2014.0898) 25540153 PMC4298196

[61] Sanderson JL , Wang J , Vitikainen EIK , Cant MA , Nichols HJ . 2015 Banded mongooses avoid inbreeding when mating with members of the same natal group. Mol. Ecol. **24** , 3738–3751. (10.1111/mec.13253) 26095171 PMC5008155

[62] Gilchrist JS . 2004 Pup escorting in the communal breeding banded mongoose: behavior, benefits, and maintenance. Behav. Ecol. **15** , 952–960. (10.1093/beheco/arh071)

[63] Kéry M , Schaub M . 2012 Bayesian population analysis using winbugs a hierarchical perspective. Waltham, MA: Academic Press.

[64] Marin X . 2021 ggmcmc: Tools for analyzing MCMC simulations from Bayesian inference. See https://cran.r-project.org/package=ggmcmc.

[65] Fasel N , Saladin V , Richner H . 2016 Alternative reproductive tactics and reproductive success in male Carollia perspicillata (Seba’s short-tailed bat). J. Evol. Biol. **29** , 2242–2255. (10.1111/jeb.12949) 27442591

[66] Freckleton RP . 2011 Dealing with collinearity in behavioural and ecological data: model averaging and the problems of measurement error. Behav. Ecol. Sociobiol. **65** , 91–101. (10.1007/s00265-010-1045-6)

[67] Youngflesh C , Che-Castaldo C , Hardy T . 2023 MCMCvis: tools to visualize, manipulate, and summarize MCMC output. See https://cran.r-project.org/package=MCMCvis.

[68] Kellner K , Meredith M . 2024 jagsUI: a wrapper around ‘rjags’ to streamline ‘JAGS’ analyses. See https://cran.r-project.org/package=jagsUI.

[69] Fichtel C , Kraus C , Ganswindt A , Heistermann M . 2007 Influence of reproductive season and rank on fecal glucocorticoid levels in free-ranging male Verreaux’s sifakas (Propithecus verreauxi). Horm. Behav. **51** , 640–648. (10.1016/j.yhbeh.2007.03.005) 17448474

[70] Young AJ , Carlson AA , Monfort SL , Russell AF , Bennett NC , Clutton-Brock T . 2006 Stress and the suppression of subordinate reproduction in cooperatively breeding meerkats. Proc. Natl Acad. Sci. USA **103** , 12005–12010. (10.1073/pnas.0510038103) 16894179 PMC1567688

[71] Cant MA , Nichols HJ , Johnstone RA , Hodge SJ . 2013 Policing of reproduction by hidden threats in a cooperative mammal. Proc. Natl Acad. Sci. **111** , 326–330. (10.1073/pnas.1312626111/-/DCSupplemental.www.pnas.org/cgi/doi/10.1073/pnas.1312626111) 24367092 PMC3890811

[72] Bell MBV , Nichols HJ , Gilchrist JS , Cant MA , Hodge SJ . 2012 The cost of dominance: suppressing subordinate reproduction affects the reproductive success of dominant female banded mongooses. Proc. R. Soc. B **279** , 619–624. (10.1098/rspb.2011.1093) PMC3234565 21752819

[73] West SA , Cooper GA , Ghoul MB , Griffin AS . 2021 Ten recent insights for our understanding of cooperation. Nat. Ecol. Evol. **5** , 419–430. (10.1038/s41559-020-01384-x) 33510431 PMC7612052

[74] Hamilton WD . 1970 Selfish and spiteful behaviour in an evolutionary model. Nature **228** , 1218–1220. (10.1038/2281218a0) 4395095

[75] Tennenhouse EM , Weladji RB , Holand Ø , Røed KH , Nieminen M . 2011 Mating group composition influences somatic costs and activity in rutting dominant male reindeer (Rangifer tarandus). Behav. Ecol. Sociobiol. **65** , 287–295. (10.1007/s00265-010-1043-8)

[76] Abell AJ . 2000 Costs of reproduction in male lizards,Sceloporus virgatus. Oikos **88** , 630–640. (10.1034/j.1600-0706.2000.880320.x)

[77] Ancona S , Drummond H , Zaldívar-Rae J . 2010 Male whiptail lizards adjust energetically costly mate guarding to male–male competition and female reproductive value. Anim. Behav. **79** , 75–82. (10.1016/j.anbehav.2009.10.005)

[78] Ritchot Y , Festa-Bianchet M , Coltman D , Pelletier F . 2021 Determinants and long-term costs of early reproduction in males of a long-lived polygynous mammal. Ecol. Evol. **11** , 6829–6845. (10.1002/ece3.7530) 34141259 PMC8207375

[79] Williams TD . 2018 Physiology, activity and costs of parental care in birds. J. Exp. Biol. **221** , jeb169433. (10.1242/jeb.169433) 30201656

[80] Zera AJ , Harshman LG . 2001 The physiology of life history trade-offs in animals. Annu. Rev. Ecol. Syst. **32** , 95–126. (10.1146/annurev.ecolsys.32.081501.114006)

[81] Salvador A , Diaz JA , Veiga JP , Bloor P , Brown RP . 2008 Correlates of reproductive success in male lizards of the alpine species Iberolacerta cyreni. Behav. Ecol. **19** , 169–176. (10.1093/beheco/arm118)

[82] Blacher P , Huggins TJ , Bourke AFG . 2017 Evolution of ageing, costs of reproduction and the fecundity–longevity trade-off in eusocial insects. Proc. R. Soc. B **284** , 20170380. (10.1098/rspb.2017.0380) PMC5524490 28701554

[83] Cox RM , Wittman TN , Calsbeek R . 2022 Reproductive trade-offs and phenotypic selection change with body condition, but not with predation regime, across island lizard populations. J. Evol. Biol. **35** , 365–378. (10.1111/jeb.13926) 34492140

[84] Firman RC , Gasparini C , Manier MK , Pizzari T . 2017 Postmating female control: 20 years of cryptic female choice. Trends Ecol. Evol. **32** , 368–382. (10.1016/j.tree.2017.02.010) 28318651 PMC5511330

[85] Gorelik G . 2021 Domains of female choice in human evolution. Evol. Behav. Sci. **17** , 187–208. (10.1037/ebs0000276)

[86] McComb KE . 1991 Female choice for high roaring rates in red deer, Cervus elaphus. Anim. Behav. **41** , 79–88. (10.1016/S0003-3472(05)80504-4)

[87] McCullough EL , Simmons LW . 2016 Selection on male physical performance during male–male competition and female choice. Behav. Ecol. **27** , 1288–1295. (10.1093/beheco/arw033)

[88] Aich U , Head ML , Fox RJ , Jennions MD . 2021 Male age alone predicts paternity success under sperm competition when effects of age and past mating effort are experimentally separated. Proc. R. Soc. B **288** , 20210979. (10.1098/rspb.2021.0979) PMC8316792 34315259

[89] Duffield KR , Bowers EK , Sakaluk SK , Sadd BM . 2017 A dynamic threshold model for terminal investment. Behav. Ecol. Sociobiol. **71** , 185. (10.1007/s00265-017-2416-z) 30002566 PMC6039117

[90] Nichols HJ , Jordan NR , Jamie GA , Cant MA , Hoffman JI . 2012 Fine-scale spatiotemporal patterns of genetic variation reflect budding dispersal coupled with strong natal philopatry in a cooperatively breeding mammal. Mol. Ecol. **21** , 5348–5362. (10.1111/mec.12015) 22994210

[91] Le Conte Y , Hefetz A . 2008 Primer pheromones in social Hymenoptera. Annu. Rev. Entomol. **53** , 523–542. (10.1146/annurev.ento.52.110405.091434) 17877458

[92] Wenseleers T , Hart AG , Ratnieks FLW . 2004 When resistance is useless: policing and the evolution of reproductive acquiescence in insect societies. Am. Nat. **164** , E154–E167. (10.1086/425223) 29641925

[93] Moore D , Liebig J . 2013 Reproductive restraint without policing in early stages of a social insect colony. Anim. Behav. **85** , 1323–1328. (10.1016/j.anbehav.2013.03.022)

[94] Smith AA , Hölldober B , Liebig J . 2009 Cuticular hydrocarbons reliably identify cheaters and allow enforcement of altruism in a social insect. Curr. Biol. **19** , 78–81. (10.1016/j.cub.2008.11.059) 19135369

[95] Bourrat P , Doulcier G , Rose CJ , Rainey PB , Hammerschmidt K . 2022 Tradeoff breaking as a model of evolutionary transitions in individuality and limits of the fitness-decoupling metaphor. eLife **11** , e73715. (10.7554/eLife.73715) 35975712 PMC9470156

[96] Birch G . 2024 Data from: Lifetime trajectories of male mating effort under reproductive conflict in a cooperatively breeding mammal. Dryad Digital Repository. (10.5061/dryad.cnp5hqcdp) PMC11513156 39288806

[97] Birch G , Nichols H , Mwanguhya F , Thompson FJ , Cant M , Blount J . 2024 Data from: Lifetime trajectories of male mating effort under reproductive conflict in a cooperatively breeding mammal. Figshare. (10.6084/m9.figshare.c.7430653) PMC11513156 39288806

